# COVID-19: Psychological distress, fear, and coping strategies among community members across the United Arab Emirates

**DOI:** 10.1371/journal.pone.0282479

**Published:** 2023-03-29

**Authors:** Rania Al Dweik, Muhammad Aziz Rahman, Fathima Mohammed Ahamed, Heba Ramada, Yousef Al Sheble, Sondos ElTaher, Wendy Cross, Deena Elsori

**Affiliations:** 1 College of Health Sciences, Abu Dhabi University, Abu Dhabi, United Arab Emirates; 2 Institute of Health and Wellbeing, Federation University Australia, Berwick, VIC, Australia; 3 Faculty of Public Health, Universitas Airlangga, Surabaya, Indonesia; 4 Al Ain Fertility Center, Abu Dhabi, United Arab Emirates; 5 School of Dentistry, Queen’s University Belfast, Belfast, United Kingdom; 6 Epidemiology Department, American University of Beirut, Beirut, Lebanon; 7 Faculty of Resilience, Rabdan Academy, Abu Dhabi, United Arab Emirates; School of Health Binawan: Universitas Binawan, INDONESIA

## Abstract

**Background:**

The COVID-19 pandemic impacted the psychosocial well-being of the United Arab Emirates [UAE] population like other communities internationally.

**Objectives:**

We aimed to identify the factors associated with psychological distress, fear, and coping amongst community members across the UAE.

**Methods:**

We conducted a cross-sectional online survey across the UAE during November 2020. Adults aged ≥18 years, living in the UAE who were able to respond to an online questionnaire in English or Arabic were considered eligible to participate in the study. We used standard validated tools to measure psychological distress, fear and coping. Kessler Psychological Distress Scale [K10] was used to assess psychological distress, Fear of COVID-19 Scale [FCV-19S] was used to assess the level of fear, and Brief Resilient Coping Scale [BRCS] was used to assess the coping strategies.

**Results:**

A total of 417 individuals participated in this study with a mean age of 29 [± 10.7] years. More than half of the participants experienced high to very high levels of psychological distress [55%] and a quarter experienced high levels of fear of COVID-19 [23.3%] with almost a third of them [36.2%] having low resilient coping. About 37.4% of the participants had work-related mental health impacts and 32.4% were perceived to have moderate to a great deal of distress due to a change of employment status during the pandemic. One in ten participants [9.4%] reported increased smoking. Increased smoking [AOR 8.66, 95% CIs 1.08–69.1,], increased alcohol drinking [AOR 2.39, 95% CIs 1.05–5.47] and higher levels of fear of COVID-19 [AOR 2.93, 95% CIs 1.83–4.67] were associated with moderate to very high levels of psychological distress. Being female [AOR 1.82, p = 0.030], having a pre-existing mental health condition [AOR 9.88, 95% p = 0.027], engaging in high-risk behaviors such as increased smoking [AOR 21.14, p = 0.003], increased alcohol drinking [AOR 1.48, p = 0.359] in the previous four weeks, and higher levels of fear of COVID-19 [AOR 4.18, p <0.001] were associated with moderate to very high levels of psychological distress. Also, being a smoker [AOR, 0.840, p = 0.011], and having a high level of fear [AOR 0.372, p = 0.001] were found to be associated with low resilient coping.

**Conclusion:**

Community members in the UAE are at a higher risk of psychosocial distress and fear during the COVID-19 pandemic. Thus, healthcare providers and policymakers would need to be more alert to provide specific mental health support strategies for their wellbeing.

## Introduction

Since the emergence of COVID-19 it has surged exponentially across the world, with more than 276 million confirmed cases and over 5.3 million deaths as of 27^th^ December 2021 [1]. The United States of America has reported the highest number of cases and deaths due to COVID-19 followed by India, Brazil, France, Russian Federation, the United Kingdom, Turkey, Italy and Spain. In Bangladesh, the first three cases of COVID-19 were detected on 8th March 2020 [24]. The United Arab Emirates [UAE] was one of the first responders to the alerts of the COVID-19 outbreak; UAE’s government, in cooperation with the National Crisis and Emergency Management Authority [NCEMA], responded before WHO considered it a pandemic [2]. In the UAE, the first cases of positive coronavirus were reported on January 23, 2020. To date 15 August 2022], there have been over **1,005,543** confirmed cases and a total of **2,339** deaths in the UAE [National Emergency Crisis and Disaster-UAE, 2022]. As of 15^th^ August 2022, a total of **24,922,054** vaccine doses have been administered in the UAE [3].

The COVID-19 pandemic brought unexpected challenges and the outbreak lead to compromised physical and mental health [3–5]. Research on past infectious disease outbreaks, such as severe acute respiratory syndrome [SARS], swine flu, and influenza revealed a wide range of psychosocial impacts at individual, community, and international levels. However, there was limited information on the mental health impact of the COVID-19 pandemic on the UAE population [6]. More recently, studies investigating the psychological impacts of COVID-19 in China, Spain, Italy, India, and the UK reported moderate to severe stress, generalized anxiety, insomnia, and depression; which were associated with lockdowns, social isolation, changes in daily habits, public fear and feelings of uncertainty [7–11]. Factors such as longer quarantine duration, fear of infection and deaths, increase in anxiety, post-traumatic stress and depression, feelings of helplessness, guilt, panic, financial loss, deaths of family members and insufficient supplies of protective equipment and tests, affected public health globally [12–15]. Regulatory laws and sudden changes turned people’s lives upside down, leaving them in shock. Available literature suggested that a psychiatric epidemic was coexisting with the COVID-19 pandemic, increasing the strain of mental health issues [16].

Economic changes indirectly added psychological distress making people more prone to mental disorders [17]. Social isolation and virtual interactions have boosted the number of people suffering from mental illnesses such as anxiety and depression in the community settings of UAE [18].

The UAE has taken unprecedented precautionary measures including complete lockdowns against COVID-19 to control its spread and ensure the well-being of individuals [19]. The UAE government launched several initiatives to combat COVID-19 including surveillance and contact tracing, containment, mental health support, mass testing and treatment, government economic support, national vaccination program. Since March 2020, wearing a face mask was mandatory, and mobile apps like the "Al Hosn" app were a must to download to aid in quarantine and track the cases. Screening programs were routinely performed to help detect new COVID-19 cases as soon as possible for an immediate medical intervention to keep their lives [2]. Compared to developed countries, the UAE has the highest number of tests conducted per 1000 people [2]. To limit the spread of COVID-19, the UAE Government introduced physical distancing rules including restrictions on social gatherings, strict lockdowns [with a pause on all social, cultural, and sporting activities], quarantine, border closures, suspension of flights, and mandating public respiratory hygiene measures [20].

The UAE’s National Program for Happiness and Wellbeing launched an online national campaign to support the UAE’s community to overcome the possible psychological impact of the pandemic COVID-19 and to provide safe and confidential mental support to individuals who are impacted directly and indirectly by COVID-19. In addition, introduced another initiative called ‘Hayat’ [Arabic word for life] is a psychological and moral support program to help federal government employees deal with the circumstances and anxiety associated with COVID-19. However, evidence-based evaluations on psychological distress, fear, and coping strategies were relatively scarce [21]. As the pandemic is still ongoing in the UAE, although it is under control, its psychological impact will not wane with its eradication. With limited research regarding the psychological distress and fear due to COVID-19 in UAE, this study aimed to investigate the factors leading to the psychological distress, the level of fear of COVID-19, and coping strategies among its population.

## Methods

### Study design and settings

This cross-sectional study was conducted across the United Arab Emirates [UAE] as part of a global study [22]. We followed the same method applied across the 17 countries in the global study. Participants were recruited using online platforms [social media and online groups] like what’s app, Facebook, Twitter, and Instagram] and were invited to answer an online survey.

### Study population

Adults aged ≥18 years, living in the UAE who were able to respond to an online questionnaire in English or Arabic were considered eligible to participate in the study. Participants included general community members, healthcare professionals, frontline or essential workers including COVID-19 hospitalized and none-hospitalized patients. Healthcare professionals and frontline or essential workers were individuals who self-identified themselves in the survey as being a doctor, nurse, or an individual in contact with patients/clients as part of their professional responsibilities during the pandemic period. Patients were defined as individuals who utilized a health care service in the last six months at the time of data collection.

### Sampling

The sample size was calculated using OpenEpi and the Snowball sampling technique was used for collecting the data. Assuming the population size is **10,069,862**, with a 50% prevalence of stress globally [no national existing data available on the prevalence of stress in the UAE], at 95% confidence intervals and 80% power, the estimated minimum sample size was 384.

### Data collection

An online survey was created using Google Forms. The survey link was made available in English with a separate link for participants who wished to take the survey in Arabic. Depending on the survey link selected by the participant, the plain language information statement [PLIS] and a consent form showed on the first screen in English or Arabic. Participants who gave their full consent and met the survey’s requirements were allowed to proceed.

Different social media channels, online community networks were used to distribute an invitation with the online survey URL and QR code, and the link to the survey was sent to staff, and students via the email databases of participating universities/hospitals, WhatsApp, and SMS text communications. The survey was open to anyone who had access to the survey URL, and no incentives were offered for taking part in the study. Participants had the option to review their responses before submitting the survey. All survey responses were anonymous.

### Study tool

The structured survey questionnaire was derived from a prior study conducted globally across 17 countries including Australia, Malaysia, and Bangladesh by the same research group [23–25]. Psychological distress was measured using the Kessler Psychological Distress Scale [K-10] [26], fear was measured using the Fear of COVID-19 Scale [FCV-19S] [27], and coping was measured using Brief Resilient Coping Scale [BRCS] [28].

The reliability of those tools in the English version was examined in the Australian study, and it was found that they worked reliably for both migrants and non-migrants [26]. We also checked the differences in reliability of the tools in English and Arabic for our study; no difference was observed. Having the multicultural population including migrants and non-migrants in the UAE, the study tools were also deemed suitable. We followed standard translation and back-translation process to create the Arabic version of the questionnaire, which was also pilot tested with necessary modifications [to clarify the languages further based on the feedback from the pilot study participants] were done as needed [24]. We used the same study tool which was used as part of the primary study in Australia [19] global study across 17 countries [24]. Study participants had the option to choose either English or Arabic version of the questionnaire while responding to this study.

### Data analyses

The database was downloaded from the Google platform and IBM SPSS Statistics 28.0 statistical software was used for data analyses. Descriptive statistics, such as means and standard deviations [SD], were generated for continuous variables; frequencies and percentages were generated for categorical variables. Fear of COVID-19 [based on the FCV-19S scoring] was categorized into low [score 7–21] and high [score 22–35], psychological distress [based on the K-10 scoring] was categorized into low [score 10–15] and moderate to very high [score 16–50], and coping [based on the BRCS scoring] was categorized into low [score 4–13] and medium to high [score 14–20]. Logistic regression analyses were used to examine the association between variables. Multivariate analyses were carried out by adjusting age, gender, smoking, alcohol intake, living status, place of birth, country, education, employment status, employment stress, healthcare worker, perceived financial impact, contact with COVID-19 case, experience due to COVID-19 [isolation/quarantine], and self-identification as a patient [who attended a clinic within last six months for any reasons]. Odds ratios [ORs] and 95% confidence intervals [CIs] were used to present the data. A cut-off of p<0.05 was considered statistically significant. For multivariate analyses, adjusted ORs [AOR] with 95% CIs were reported.

### Ethics approval and consent to participate

Ethics approval was obtained from Abu Dhabi University Institutional Research Board [Ref: CoHS– 20-10-00024]. The survey was voluntary, and it was explained in plain English and Arabic, so that participants could make an informed decision about whether or not to participate in the study. The survey was anonymous and the data were handled only by the investigators listed in the study.

## Results

A total of 417 individuals participated in this study and more than two-thirds of them [291, 70%] responded in English. The majority of them lived with family members [381, 91.4%] and belonged to the age group of 18–29 years [254, 60.9%]. Less than half of the participants [182, 43.6%] were born in the UAE and more than half of the study population [240, 57.6%] completed at least a bachelor’s degree. Less than half of participants [156, 37.4%] lose/ reduced working hours/or were afraid to lose their jobs during this pandemic, and 32.4% [135] were perceived to have moderate to a great deal of distress due to a change of employment status due to pandemic. COVID-19 did not impact the financial situation of more than half of the participants [224,53.7%]. Only 13.4% [56] identified themselves as frontline or essential service workers, including doctors [1.7%], nurses [1%], and other healthcare professionals [6.2%] [Table 1]. More than half of the study participants [302,72.4%] did not report any comorbidity. However, 8.2% [34] reported having pre-existing psychiatric or mental health issues and 19.4% [81] reported other comorbidities conditions. More than half of the participants were never smoked or drink alcohol [71.9%, and 89% respectively]. However, of those who smoked, 9.4% [39] reported increased smoking in the previous four weeks [Table 1].

**Table 1:** Characteristic of the study population in UAE [November-2020].

Characteristics	Total n [%]
**Total study participants**	**417**
**Age groups**	**405**
18–29 years	254 [60.9]
30–59 years	149 [35.7]
60+ years	2 [0.48]
**Gender**	**414**
Male	120 [28.8]
Female	294 [70.5]
**Living Status**	**412**
Live without family members [on your own/shared house/others]	31 [7.4]
Live with family members [partner and/or children]	381 [91.4]
**Born in UAE**	**415**
No	233 [55.9]
Yes	182 [43.6]
**Completed level of education**	**414**
Grade 1–12	125 [30.5]
Trade/Certificate/Diploma	49 [11.8]
Bachelor and above	240 [57.6]
**Current employment condition**	**399**
Jobs affected by COVID-19 [lost job/working hours reduced/afraid of job loss]	156 [37.4]
Have an income source [employed/Government benefits]	243 [58.3]
**Perceived distress due to change of employment status**	**367**
A little to none	232 [55.6]
Moderate to a great deal	135 [32.4]
**Self-identification as frontline or essential service worker**	**417**
Yes	56 [13.4]
No	361 [86.6]
**COVID-19 Impacted financial situation**	**417**
Yes, impacted positively	36 [8.6]
Yes, impacted negatively	157 [37.6]
No impact	224 [53.7]
**Co-morbidities**	**417**
No comorbid conditions	302[72.4]
Psychiatric/ Mental health issues	34 [8.2]
Other comorbid conditions	81 [19.4]
**Smoking**	**417**
Never smoker	300[71.9]
Ex-smoker	40 [9.6]
Occasional smoker	16 [3.8]
Less than weekly, but at least once a month	10 [2.4]
Less than daily, but at least once a week	11 [2.6]
Daily	40 [9.6]
**Increase smoking over the last 6 months**	**77**
Yes	39 [9.4]
No	38 [9.1]
**Current alcohol drinking**	**371**
Yes	39 [9.4]
No	371 [89.0]
**Increased alcohol drinking over the last 6 months**	**39**
Yes	16 [3.8]
No	23 [5.5]
**Provide care to a family member/patient with a known/suspected case of COVID-19**	**410**
No	285 [68.3]
Unsure	32 [7.7]
Yes	93[22.7]
**Experience related to COVID-19 pandemic [multiple responses possible]**	**405**
No known exposure to COVID-19	336 [80.6]
Tested positive for COVID-19	16 [3.8]
Tested negative for COVID -19 but self-isolating	42 [10.1]
Recent overseas travel history and was in quarantine	11 [2.6]
**Health Service use in the last 4 weeks**	**405**
Yes	205 [48.8]
No	200 [49.2]
**Type of health service users to overcome COVID-19 related stress in the last 4 weeks**	**30**
Consult a GP	12 [2.9]
Consulted a psychologist	3 [0.7]
Consulted a psychiatrist	1 [0.2]
Used mental health resources available in media	9 [2.2]
Used mental health support services	1 [0.2]
Used combination of services	4 [1.0]

22.7% [93] of the participants provided direct care to family members or patients with a known or suspected case of COVID-19. Almost half of the participants [205, 48.8%] used health services in the previous four weeks.

More than two-thirds of the participants [328, 78.8%] experienced moderate to very high levels of psychological distress, 23.3% [97] had high levels of fear of COVID-19, and more than half of the participants [262, 62.8%] had medium to high resilient coping [S1-S3 Tables in S1 File].

### Psychological distress

The results in Table 2 show the factors associated with moderate to very high psychological distress among the participants. Younger participants, females, participants living family members, having graduated, employed, those with pre-existing mental health conditions, did not identify themselves as frontline workers, increased smoking and alcohol drinking in the last four weeks, self-isolating, used health service in general, or used health service to overcome COVID-19 related stress in the last four weeks, and those with a higher level of fear of COVID-19 were more likely to develop moderate to very high levels of psychological distress compared to their counterparts [Table 4]. However, when potential confounders were adjusted, being female [AOR 1.82, p = 0.030], having a pre-existing mental health condition [AOR 9.88, 95% p = 0.027], and engaging in high-risk behaviors such as increased smoking [AOR 21.14, p = 0.003] in the previous four weeks, and higher levels of fear of COVID-19 [AOR 4.18, p <0.001] were associated with moderate to very high levels of psychological distress. However, living with family members [AOR 0.487, p = 0.184] and having medium to high resilient coping [AOR 0.386, p = 0.002] were associated with low levels of psychological distress [Table 2].

**Table 2:** Factors related to high psychological distress among the study population [based on K10 score] in UAE [November-2020].

Characteristics	Moderate to Very High [Score 16–50], n [%]	Low [Score 10–15], n [%]	*p*	OR	95% Cl	p	AOR	95%Cl
Total study participants	**319**	**86**						
Age groups	**319**	**86**						
**18–29 years**	222	32		1			1	
**30–59 years**	96	53	<0.001	0.261	0.158–0.430	<0.001	0.305	0.152–0.614
**60+ years**	1	1	0.175	0.144	0.009–2.362	0.284	0.200	0.011–3.799
Gender	**326**	**88**						
**Male**	82	38		1			1	
**Female**	244	50	0.001	2.260	1.385–3.696	0.030	1.828	1.060–3.152
Living Status	**324**	**88**						
**Live without family members [on your own/shared house/others]**	26	5		1			1	
**Live with family members [partner and/or children]**	298	83	0.462	0.690	0.257–1.854	0.242	0.529	0.182–1.536
Born in UAE	**327**	**88**						
**No**	175	58		1			1	
**Yes**	152	30	0.039	1.679	1.027–2.745	0.889	1.046	0.558–1.959
Completed level of education	**326**	**88**						
**Grade 1 to 6 Primary**	**1**	**0**		**1**			**1**	
**Grade 7 to 12**	106	18	0.388	2.944	0.254–34.186	0.332	3.374	0.289–39.348
**Certificate/Diploma**	46	3	0.135	7.667	0.531–110.650	0.077	11.308	0.770–166.070
**Degree [Bachelor/Masters or above]**	173	67	0.836	1.291	0.115–14.475	0.416	2.767	0.238–32.179
Current employment condition	**328**	**89**						
**Unemployed/Home duties**	**16**	**2**		**1**			**1**	
**Jobs affected by COVID-19 [lost job/working hours reduced/afraid of job loss]**	110	46	0.240	0.399	0.086–1.852	0.637	0.594	0.068–5.161
**Have an income source [employed/Government benefits]**	202	41	0.801	0.821	0.177–3.808	0.645	0.609	0.074–5.000
**Perceived distress due to change of employment status**	293	74						
**A little to None**	**169**	**63**		**1**			**1**	
**Moderate to a great deal**	**124**	**11**	**<0.001**	**4.202**	**2.127–8.304**	**<0.001**	**4.379**	**2.018–9.582**
Self-identification as frontline or essential service worker	**328**	**89**						
**No**	286	75		1			1	
**Yes**	42	14	0.474	0.787	0.408–1.516	0.863	1.081	0.500–2.338
COVID-19 impacted the financial situation	**328**	**89**						
**No**	160	64		1			1	
**Yes**	168	25	<0.001	4.086	2.196–7.600	<0.001	5.099	1.966–13.224
Co-morbidities	**328**	**89**						
**No**	232	70		1			1	
**Psychiatric/Mental health problem**	33	1	0.025	9.957	1.338–74.112	0.038	8.673	1.129–66.623
**Other co-morbidities**	63	18	0.856	1.056	0.587–1.901	0.038	2.094	1.042–4.207
Smoking	**328**	**89**						
**Never smoker**	239	61		1			1	
**Ever smoker**	89	28	0.873	0.988	0.856–1.141	0.732	0.906	0.515–1.594
Increased smoking in the last 4 weeks	**62**	**15**						
**No**	25	13		1			1	
**Yes**	37	2	0.005	9.620	1.996–46.370	0.003	61.006	4.063–915.988
Current alcohol drinking [last 4 weeks]	**323**	**87**						
**No**	293	78		1			1	
**Yes**	30	9	0.766	0.887	0.404–1.947	0.345	1.509	0.642–3.546
Increases alcohol drinking over the last 6 months	**30**	**10**						
**No**	15	9		1			1	
**Yes**	15	1	0.064	8.00	0.888–72.099	0.044	1.487	0.637–88.545
Provide care to a family member/patient with a known/suspected case of COVID-19	**322**	**88**						
**No**	218	67		1			1	
**Yes**	104	21	0.194	1.193	0.914–1.558	0.543	0.680	0.197–2.351
Experience related COVID-19 pandemic	**316**	**89**						
**No known exposure to COVID-19**	**257**	**79**		**1**			**1**	
**I had been tested negative for COVID-19 but self-isolating**	**36**	**6**	**0.192**	**1.821**	**0.740–4.482**	**0.212**	**1.8.23**	**0.710–4.684**
**I had been tested positive for COVID-19**	**16**	**0**	**0.323**	**0.531**	**0.152–1.862**	**0.125**	**0.286**	**0.058–1.413**
Self-identification as a patient/Use of health service in the last 4 weeks	**319**	**86**						
**No**	**151**	**49**		**1**			**1**	
**Yes**	**168**	**37**	0.114	1.473	0.912–2.381	**0.091**	**1.588**	**0.929–2.713**
Healthcare service use in the last 6 months	**162**	**36**						
**Telehealth consultation/Used helpline**	**14**	**1**		**1**			**1**	
**Visited a healthcare provider in person**	**129**	**30**	**0.263**	**3.256**	**0.412–25.732**	**0.906**	**1.149**	**0.114–11.544**
**Used both services**	**19**	**5**	**0.820**	**0.884**	**0.305–2.556**	**0.330**	**0.518**	**0.138–1.945**
Level of fear of COVID-19 [FCV-19S categories]	**328**	**89**						
**Low [score 7–21]**	**240**	**80**		**1**			**1**	
**High [score 22–35]**	**88**	**9**	**0.002**	**3.259**	**1.569–6.771**	**0.001**	**4.148**	**1.766–9.741**
Level of coping [BRCS categories]	**324**	**89**						
**Low resilient coping [score 4–13]**	**130**	**21**		**1**			**1**	
**Medium to high resilient coping [score 14–20]**	**194**	**68**	**0.005**	**0.461**	**0.269–0.789**	**0.002**	**0.383**	**0.208–0.704**
Healthcare service use to overcome COVID-19 related stress in the last 4 weeks	**316**	**89**						
**No**	**281**	**88**		**1**			**1**	
**Yes**	**35**	**1**	**0.019**	**10.961**	**1.48–81.16**	**0.016**	**12.134**	**1.596–92.249**

Adjusted for: age, gender, living status, residence location, born in UAE, education and employment.

### Fear of COVID-19

The levels of fear of COVID-19 were associated with several factors following the adjustment of potential confounders. Higher levels of fear from COVID -19 were associated with factors such as: having a Bachelors and Masters level of education or above [AOR 4.14, p = 0.011], perceived distress due to changes in employment status [AOR 2.06, p = 0.009], impacted financial situation due to COVID-19 [AOR 1.38, p = 0.013], having pre-existing mental health issues or existing psychiatric problems [AOR 2.37, p = 0.030], and having moderate to a very high level of psychological distress [AOR 4.09, p = 0.001] [Table 3].

**Table 3:** Factors associated with levels of fear of COVID-19 among the study population [based on FCV-19S score] in UAE [November-2020].

Characteristics	High [Score 22–35], n [%]	Low [Score 7–21], n [%]	*p*	OR	95% Cl	p	AOR	95%Cl
**Total study participants**	**94**	**311**						
**Age groups**	**94**	**311**						
18–29 years	63	191		**1**			**1**	
30–59 years	30	119	0.284	0.764	0.468–1.249	0.996	0.986	0.508–1.913
60+ years	1	1	0.435	3.032	0.187–49.182	0.333	4.175	0.231–75.333
**Gender**	**95**	**319**						
Male	24	96		1			1	
Female	71	223	0.363	1.274	0.756–2.144	0.470	1.229	0.702–2.150
**Living Status**	**96**	**316**						
Live without family members [on your own/shared house/others]	8	23		1			1	
Live with family members [partner and/or children]	88	293	0.732	0.863	0.373–1.998	0.644	0.812	0.335–1.965
**Born in UAE**	**97**	**318**						
No	52	181		1			1	
Yes	45	137	0.565	1.143	0.724–1.805	0.961	0.987	0.569–1.710
**Current employment condition**	**97**	**320**						
Unemployed/Home duties	4	14	0.935	0.946	0.249–3.594	0.621	1.516	0.291–7.899
Jobs affected by COVID-19 [lost job/working hours reduced/afraid of job loss]	32	124	0.758	1.229	0.332–4.551	0.623	1.483	0.308–7.137
Have an income source [employed/Government benefits]	61	182	0.880	1.222	0.091–16.429	0.886	1.270	0.080–20.217
**Perceived distress due to change of employment status**	**80**	**287**						
A little to None	41	191		1			1	
Moderate to a great deal	39	96	0.013	1.893	1.145–3.127	0.009	2.062	1.203–3.536
**Self-identification as frontline or essential service worker**	**97**	**320**						
No	86	275		1			1	
Yes	11	45	0.492	0.782	0.387–1.578	0.930	0.967	0.456–2.049
**COVID-19 impacted the financial situation**	**97**	**320**						
No	43	181		1			1	
Yes	54	139	0.017	1.340	1.055–1.703	0.012	1.912	1.151–3.175
**Co-morbidities**	**97**	**320**						
No	64	238		1			1	
Psychiatric/Mental health problem	14	20	0.011	2.603	1.246–5.438	0.030	2.375	1.089–5.176
Other co-morbidities	19	62	0.661	1.140	0.636–2.042	0.994	1.002	0.521–1.930
**Smoking**	**97**	**320**						
Never smoker	73	227		1			1	
Ever smoker	24	93	0.693	0.971	0.841–1.122	0.470	0.814	0.465–1.423
**Increased smoking in the last 4 weeks**	**15**	**62**						
No	2	36		1			1	
Yes	13	26	0.006	9.00	1.869–43.34	0.031	6.50	1.191–35.486
**Current alcohol drinking [last 4 weeks]**	**94**	**316**						
No	85	286		1			1	
Yes	9	30	0.981	1.009	0.461–2.209	0.929	1.039	0.451–2.390
**Increases alcohol drinking over the last 4 weeks**	**9**	**30**						
No	5	19		1			1	
Yes	4	11	0.812	1.200	0.267–5.400	0.194	4.213	0.480–36.978
**Provide care to a family member/patient with a known/suspected case of COVID-19**	**95**	**315**						
No	63	222		1			1	
Yes	32	93	0.747	1.040	0.819–1.321	0.201	0.369	0.080–1.701
**Experience related COVID-19 pandemic**	**94**	**311**						
No known exposure to COVID-19	86	250		1			1	
I had been tested positive for COVID-19	1	15	0.100	0.181	0.024–1.385	0.584	1.280	0.529–3.095
I had been tested negative for COVID-19 but self-isolating	7	35	0.207	0.579	0.248–1.352	0.209	1.476	0.805–2.706
I had recent overseas travel history and was in self-quarantine	0	11	0.999			0.201	0.369	0.080–1.701
**Self-identification as a patient/Use of health service in the last 4 weeks**	**94**	**311**						
No	42	158		1			1	
Yes	52	153	0.299	1.279	0.804–2.032	0.404	1.232	0.754–2.014
**Healthcare service use in the last 4 weeks**	**49**	**149**						
Telehealth consultation/Used helpline	3	12		1			1	
Visited a healthcare provider in person	22	126	0.945	0.955	0.255–3.580	0.287	0.316	0.038–2.635
Used both services	13	11	<0.001	4.512	1.854–10.985	0.014	3.383	1.284–8.914
**Level of psychological distress [K10 categories]**	**97**	**320**						
Low [score 10–15]	9	80		1			1	
Moderate to Very high [score 16–50]	88	240	0.002	3.259	1.569–6.771	0.001	4.093	1.754–9.548
**Level of coping [BRCS categories]**	**93**	**320**						
Low resilient coping [score 4–13]	37	114		1			1	
Medium to high resilient coping [score 14–20]	56	206	0.464	0.838	0.521–1.346	0.568	0.862	0.518–1.434
**Healthcare service use to overcome COVID-19 related stress in the last 6 months**	**92**	**313**						
No	76	293		1			1	
Yes	16	20	0.002	3.084	1.525–6.237	<0.001	3.582	1.707–7.516

### Coping strategies

The multivariate analyses showed that there was no significant difference between those with medium to high resilient coping when compared to those with low resilient coping based on the BRCS scale. On the other hand, being a smoker [AOR, 0.840, p = 0.011], and having a high level of fear [AOR 0.372, p = 0.001] were found to be associated with low resilient coping [Table 4].

**Table 4:** Factors associated with medium to high resilience coping among the study participants [based on BRCS score] in UAE [November-2020].

Characteristics	Medium to High [Score 14–20], n [%]	Low [Score 4–13], n [%]	*p*	OR	95% Cl	p	AOR	95%Cl
**Total study participants**	**257**	**144**						
**Age groups**	**257**	**144**						
18–29 years	149	101		1			1	
30–59 years	106	43	0.021	1.671	1.081–2.582	0.275	1.386	0.771–2.490
60+ years	2	0				0.999		
**Gender**	**260**	**150**						
Male	75	45		1			1	
Female	185	105	0.805	1.057	0.681–1.642	0.290	1.297	0.801–2.099
**Living Status**	**257**	**151**						
Live without family members [on your own/shared house/others]	23	8		1			1	
Live with family members [partner and/or children]	234	143	0.184	0.569	0.248–1.307	0.520	0.751	0.314–1.795
**Born in UAE**	**262**	**149**						
No	163	70		1			1	
Yes	99	79	0.003	0.538	0.358–0.809	0.090	0.655	0.402–1.068
**Current employment condition**	**262**	**151**						
Unemployed/Home duties	16	2		1			1	
Jobs affected by COVID-19 [lost job/working hours reduced/afraid of job loss]	106	50	0.184	0.353	0.076–1.639	0.091	0.161	0.019–1.339
Have an income source [employed/Government benefits]	140	99	0.062	0.236	0.052–1.076	0.46	0.120	0.015–0.960
**Perceived distress due to change of employment status**	**227**	**140**						
A little to None	145	87		1			1	
Moderate to a great deal	82	53	0.738	0.928	0.600–1.435	0.858	1.044	0.648–1.682
**Self-identification as frontline or essential service worker**	**262**	**151**						
No	227	130		1			1	
Yes	35	21	0.875	0.954	0.533–1.709	0.707	0.882	0.459–1.695
**COVID-19 impacted the financial situation**	**262**	**151**						
No	140	80		1			1	
Yes	122	71	0.831	1.023	0.827–1.266	0.410	1.216	0.763–1.939
**Co-morbidities**	**262**	**151**						
No	197	105		1			1	
Psychiatric/Mental health problem	15	15	0.102	0.533	0.251–1.133	0.084	0.494	0.222–1.100
Other co-morbidities	50	31	0.559	0.860	0.518–1.427	0.992	0.997	0.570–1.745
**Smoking**	**262**	**151**						
Never smoker	201	95		1			1	
Ever smoker	61	56	0.022	0.869	0.770–0.980	0.001	0.432	0.267–0.701
**Increased smoking in the last 6 months**	**36**	**41**						
No	25	13		1			1	
Yes	16	23	0.031	0.362	0.143–0.913	0.222	0.504	0.168–1.513
**Current alcohol drinking [last 4 weeks]**	**255**	**151**						
No	228	139		1			1	
Yes	27	12	0.384	1.372	0.673–1.123	0.967	0.984	0.460–2.103
**Increases alcohol drinking over the last 6 months**	**27**	**12**						
No	15	9		1			1	
Yes	12	3	0.249	2.40	0.530–10.877	0.768	0.731	0.091–5.895
**Provide care to a family member/patient with a known/suspected case of COVID-19**	**258**	**148**						
No	176	109		1			1	
Yes	82	29				0.737	1.041	0.825–1.312
**Experience related COVID-19 pandemic**	**254**	**147**						
No known exposure to COVID-19	217	118		1			1	
Tested positive for COVID-19	10	6	0.873	0.919	0.326–2.592	0.469	0.646	0.198–2.109
Tested negative for COVID-19 but self-isolating	20	22	0.036	0.501	0.263–0.956	0.024	0.458	0.233–0.901
Recent overseas travel history and was in self-quarantine	10	1	0.106	5.514	0.697–43.605	0.455	2.280	0.262–19.842
**Self-identification as a patient [visited a healthcare provider in the last 4 weeks]**	**257**	**144**						
No	122	78		1			1	
Yes	135	66	0.199	1.308	0.869–1.969	0.300	1.260	0.813–1.953
**Healthcare service use in the last 4 weeks**	**131**	**63**						
Telehealth consultation/Used helpline	7	8		1			1	
Visited a healthcare provider in person	108	51	0.105	0.413	0.142–1.202	0.733	0.812	0.245–2.690
Used both services	16	4	0.276	1.899	0.601–5.937	0.130	2.794	0.738–10.585
**Level of fear of COVID-19 [K10 categories]**	**262**	**151**						
Low [score 16–15]	68	21		1			1	
Moderate to Very High [score 16–50]	194	130	0.005	0.461	0.269–0.789	0.001	0.372	0.202–0.684
**Level of fear of COVID-19 [FCV-19S categories]**	**262**	**151**						
Low [score 10–15]	206	114		1			1	
High [score 16–50]	56	37	0.464	0.838	0.521–1.346	0.576	0.865	0.519–1.440
**Healthcare service use to overcome COVID-19 related stress in the last 4 weeks**	**257**	**148**						
No	230	139		1			1	
Yes	27	9	0.136	1.813	0.828–3.968	0.193	1.716	0.761–3.872

## Discussion

This study is one of the first studies in identifying factors related to psychological distress, fear, and coping among the UAE residents during the COVID-19 pandemic.

During an outbreak of infection, previous research has demonstrated a wide range of psychosocial effects on people at the individual and communal levels [29]. The results of this study showed that around 78.7% of respondents experienced moderate to very high levels of psychological distress which was higher than the findings from similar studies on psychological distress during the pandemic in Australia [62.6%], Bangladesh [69%], Malaysia [62.1%] and Saudi Arabia [72%] and China [53.8%] [28, 38–42] [24]

According to a previous study, patients with a history of smoking are more likely to develop severe COVID disease and be admitted to critical care [30]. This study found a significant association between increased alcohol consumption and smoking in the previous six months and higher psychological distress. However, we are not able to establish temporal relationship due to the nature of cross-sectional study design.

Evidence suggests that practicing social distancing poses a challenge to the mental health of all family members of multiple generations living together because there was decreased social support through family interaction and cultural activities, which led to feelings of loneliness, negative emotions, and psychological distress [31]. In addition, members experienced fear of infections for themselves or their family, anxiety, fear of death, and other mental health concerns [32]. Studies demonstrated a significant negative impact of fear of COVID-19 on mental health leading to depression, anxiety, or stress [33]. Fear of COVID-19 has also been found to be associated with decreased life satisfaction with increased mental health challenges in the context of the current pandemic [34–36].

The results of this study were consistent with the results from other countries including the US, Australia, and Bangladesh where females and those with pre-existing mental health conditions reported higher psychiatric distress [23, 24, 37]. There are a variety of reasons for this, including the fact that women disproportionately share the majority of caregiving tasks in both the formal and informal sectors. They are also more frequently the primary caregivers in a household, which may exacerbate their anxiety and stress in a pandemic situation [38]

Higher levels of fear in this study were significantly associated with distress due to employment status, impact on the financial situation, co-morbidity including psychiatric and mental health problems, and moderate to very high psychological distress. Similar results were reported in both Australian and Bangladesh results [23, 24]. Fear of contracting the infection, quarantine measures for infected individuals, and self-isolation/social distancing for the general population may have played a major role in influencing their mental health during the critical phases of the pandemic and could explain this association [39, 40]. A study reported that patients affected with COVID-19 had a high level of post-traumatic stress symptoms and a significantly higher level of depressive symptoms [41].

This study found that 62.8% of the participants had high resilient coping. These findings were similar to the results from the global study [57%] and Bangladesh study [57.1%] [22, 33]. This disparity could be explained by people learning from previous success experiences, allowing them to cope better [42].

The UAE Ministry of Health introduced psychological and moral support program called Hayat’ [Arabic word for life]to help federal government employees deal with the circumstances and anxiety associated with COVID-19 [23].

## Strengths

The use of validated measures to evaluate the elements associated with psychological distress, fear, and coping methods among a large number of UAE residents during the COVID-19 pandemic and the achievement of the target sample size was considered as strengths of the study. Another strength was achieving the target sample size within all COVID-19 restrictions.

## Limitations

Since this was an online survey, the majority of the responses were from the age group 18 to 29 years, implying that they were more active on social media and had more online access. The use of the snowball sampling technique may have resulted in selection bias, and the survey’s self-reporting nature may have resulted in reporting bias. However, due to the restrictions on movement at the time of the pandemic, such sampling was deemed viable. Data were collected in late 2020, so the habituation effects could not be ruled out.

## Conclusion

The COVID-19 pandemic had an adverse impact on mental health in the UAE community. The UAE government has taken unprecedented precautionary measures including complete lockdowns against COVID-19 to control its spread and ensure the well-being of individuals. The findings of this study showed that people with mental illnesses, females, and frontline workers were at high risk of fear and psychological distress. In general, the COVID-19 pandemic had a major negative impact on public mental health. Healthcare authorities in collaboration with various sectors are recommended to apply for psychological help and design appropriate educational programs to improve the mental health of the public.

## Supporting information

S1 File (DOCX)

## Abbreviations

AOR: Adjusted Odds Ratio
BRCS: Brief Resilient Coping Scale
Cis: Confidence Interval
FCV-19S: Fear of COVID-19 Scale
K-10: Kessler Psychological Distress Scale
ORs: Odds Ratios
PCR: Polymerase Chain Reaction
