# Supplementary material for: COVID-19: Psychological distress, fear, and coping strategies among community members across the United Arab Emirates
Source: PLoS One. 2023 Mar 29;18(3):e0282479. doi: 10.1371/journal.pone.0282479 (PMC10057807; doi:10.1371/journal.pone.0282479)
Supplement: S1 File — (DOCX) [file pone.0282479.s001.docx]

**Supplementary tables:**

**Supplementary Table 1: Level of Psychological distress among the study participants**

| **Anxiety and Depression Checklist [K10] [last week]** | **Total n [%]** |
| --- | --- |
| **About how often did you feel tired out for no good reason?** | **417** |
| None | 71 [17.0] |
| A little of the time | 94 [22.5] |
| Some of the time | 134 [32.1] |
| Most of the time | 91[21.8] |
| All the time | 27 [6.5] |
| **How often did you feel nervous?** | **417** |
| None | **60 [14.4]** |
| A little of the time | **90 [21.6]** |
| Some of the time | **117 [28.1]** |
| Most of the time | **95 [22.8]** |
| All the time | **55 [13.2]** |
| **How often did you feel so nervous that nothing could calm you down?** | **417** |
| None | **141 [33.8]** |
| A little of the time | **111 [26.6]** |
| Some of the time | **95 [22.8]** |
| Most of the time | **54 [12.9]** |
| All the time | **16 [3.8]** |
| **How often did you feel hopeless?** | **417** |
| None | **142 [34.1]** |
| A little of the time | **94 [22.5]** |
| Some of the time | **90 [21.6]** |
| Most of the time | **68 [16.3]** |
| All the time | **23 [5.5]** |
| **How often did you feel restless or fidgety?** | **417** |
| None | **80 [19.2]** |
| A little of the time | **112 [26.9]** |
| Some of the time | **109 [26.1]** |
| Most of the time | **85 [20.4]** |
| All the time | **31 [7.4]** |
| **How often did you feel so restless you could not sit still?** | **417** |
| None | **174 [41.7]** |
| A little of the time | **99 [23.7]** |
| Some of the time | **97 [23.3]** |
| Most of the time | **33 [7.9]** |
| All the time | **14 [3.4]** |
| **How often did you feel so depressed?** | **417** |
| None | **129 [30.9]** |
| A little of the time | **90 [21.6]** |
| Some of the time | **114 [27.3]** |
| Most of the time | **62 [14.9]** |
| All the time | **22 [5.3]** |
| **How often did you feel that everything was an effort?** | **417** |
| None | **83 [19.9]** |
| A little of the time | **105 [25.2]** |
| Some of the time | **123 [29.5]** |
| Most of the time | **61 [14.6]** |
| All the time | **45 [10.8]** |
| **How often did you feel so sad that nothing could cheer you up?** | **417** |
| None | **139 [33.3]** |
| A little of the time | **101 [24.2]** |
| Some of the time | **91 [21.8]** |
| Most of the time | **63 [15.1]** |
| All the time | **23 [5.5]** |
| **How often did you feel worthless?** | **417** |
| None | **193 [46.3]** |
| A little of the time | **99 [23.7]** |
| Some of the time | **53 [12.7]** |
| Most of the time | **43 [10.3]** |
| All the time | **29 [7.0]** |
| **Level of psychological distress [K10 categories]** | **417** |
| **Low [Score 10 – 15]** | **89 [21.3]** |
| **Moderate to Very High [Score 16-50]** | **328 [78.7]** |

**Supplementary Table 2: Level of fear of COVID-19 among the study participants**

| **Fear of COVID-19 Scale [FCV -195] individual items** | **Total n [%]** |
| --- | --- |
| **I am most afraid of COVID -19** | **417** |
| Strongly disagree | 66 [15.8] |
| Somewhat disagree | 59 [14.1] |
| Neither agree nor disagree | 112 [26.9] |
| Somewhat agree | 125 [30] |
| Strongly agree | 55 [13.2] |
| **It makes me uncomfortable to think about COVID-19** | **417** |
| Strongly disagree | **80 [19.2]** |
| Somewhat disagree | **60 [14.4]** |
| Neither agree nor disagree | **94 [22.5]** |
| Somewhat agree | **118 [28.3]** |
| Strongly agree | **65 [15.6]** |
| **My hands become clammy when I think about COVID-19** | **417** |
| Strongly disagree | **225 [54.0]** |
| Somewhat disagree | **81 [19.4]** |
| Neither agree nor disagree | **80 [19.12]** |
| Somewhat agree | **21 [5.0]** |
| Strongly agree | **10 [2.4]** |
| **I am afraid of losing my life because of COVID-19** | **417** |
| Strongly disagree | **153 [36.7]** |
| Somewhat disagree | **61 [14.6]** |
| Neither agree nor disagree | **85 [20.4]** |
| Somewhat agree | **83 [19.9]** |
| Strongly agree | **35 [8.4]** |
| **When watching news and stories about COVID-19 on social media, I become nervous or anxious** | **417** |
| Strongly disagree | 88 [21.1] |
| Somewhat disagree | 64 [15.3] |
| Neither agree nor disagree | 96 [23.0] |
| Somewhat agree | 127 [30.5] |
| Strongly agree | 42 [10.1] |
| **I cannot sleep because I’m worried about getting COVID-19** | **417** |
| Strongly disagree | **258 [61.9]** |
| Somewhat disagree | **80 [19.2]** |
| Neither agree nor disagree | **54 [12.9]** |
| Somewhat agree | **20 [4.8]** |
| Strongly agree | **5 [1.2]** |
| **My heart races or palpitates when I think about getting COVID-19** | **417** |
| Strongly disagree | 231 [55.4] |
| Somewhat disagree | 66 [15.8] |
| Neither agree nor disagree | 67 [16.1] |
| Somewhat agree | 45 [10.8] |
| Strongly agree | 8 [1.9] |
| **Level of fear of COVID-19 [FCV -195 categories]** | **417** |
| Low [Score 7 -21] | 320 [76.7] |
| High [Score 22 -35] | 97 [23.3] |

**Supplementary Table 3: Coping during COVID-10 pandemic among the study participants**

| **Brief Resilient Coping Scale [BRCS] individual items** | **417** |
| --- | --- |
| Does not describe me at all | 15 [3.6] |
| Does not describe me | 37 [8.9] |
| Neutral | 144 [34.5] |
| Describes me | 154 [36.9] |
| Describes me very well | 63 [15.1] |
| **Regardless of what happens to me, I believe I can control my reaction to it** | **413** |
| Does not describe me at all | 15 [3.6] |
| Does not describe me | 42 [10.1] |
| Neutral | 113 [27.1] |
| Describes me | 182 [43.6] |
| Describes me very well | 61 [14.6] |
| **I believe I can grow in positive ways by dealing with difficult situations** | **413** |
| Does not describe me at all | 5 [1.2] |
| Does not describe me | 24 [5.8] |
| Neutral | 109 [26.1] |
| Describes me | 171[24.9] |
| Describes me very well | 104 [24.9] |
| **I actively look for ways to replace the losses I encounter in life** | **413** |
| Does not describe me at all | 9 [2.2] |
| Does not describe me | 43 [10.3] |
| Neutral | 131 [31.4] |
| Describes me | 163 [39.1] |
| Describes me very well | 67 [16.1] |
| **Level of coping [BRCS categories]** | **413** |
| Low resilient coping [score 4- 13] | 151 [36.2] |
| Medium to High resilient coping [score 14- 16] | 262 [62.8] |
